# Role of the GLP2–Wnt1 axis in silicon-rich alkaline mineral water maintaining intestinal epithelium regeneration in piglets under early-life stress

**DOI:** 10.1007/s00018-024-05162-x

**Published:** 2024-03-12

**Authors:** Jian Chen, Xue-Yan Dai, Bi-Chen Zhao, Xiang-Wen Xu, Jian-Xun Kang, Ya-Ru Xu, Jin-Long Li

**Affiliations:** 1 College of Veterinary Medicine, Northeast Agricultural University, Harbin, 150030 People’s Republic of China; 2 Key Laboratory of the Provincial Education, Department of Heilongjiang for Common Animal Disease Prevention and Treatment, Northeast Agricultural University, Harbin, 150030 People’s Republic of China; 3 Heilongjiang Key Laboratory for Laboratory Animals and Comparative Medicine, Northeast Agricultural University, Harbin, 150030 People’s Republic of China

**Keywords:** Maternal separation piglet model, Intestinal epithelial injury, Cell cycle, Intestinal stem cell, G1–S-phase checkpoint

## Abstract

Stress-induced intestinal epithelial injury (IEI) and a delay in repair in infancy are predisposing factors for refractory gut diseases in adulthood, such as irritable bowel syndrome (IBS). Hence, it is necessary to develop appropriate mitigation methods for mammals when experiencing early-life stress (ELS). Weaning, as we all know, is a vital procedure that all mammalian newborns, including humans, must go through. Maternal separation (MS) stress in infancy (regarded as weaning stress in animal science) is a commonly used ELS paradigm. Drinking silicon-rich alkaline mineral water (AMW) has a therapeutic effect on enteric disease, but the specific mechanisms involved have not been reported. Herein, we discover the molecular mechanism by which silicon-rich AMW repairs ELS-induced IEI by maintaining intestinal stem cell (ISC) proliferation and differentiation through the glucagon-like peptide (GLP)2–Wnt1 axis. Mechanistic study showed that silicon-rich AMW activates GLP2-dependent Wnt1/β-catenin pathway, and drives ISC proliferation and differentiation by stimulating Lgr5^+^ ISC cell cycle passage through the G1–S-phase checkpoint, thereby maintaining intestinal epithelial regeneration and IEI repair. Using GLP2 antagonists (GLP2^3−33^) and small interfering RNA (SiWnt1) in vitro, we found that the GLP2–Wnt1 axis is the target of silicon-rich AMW to promote intestinal epithelium regeneration. Therefore, silicon-rich AMW maintains intestinal epithelium regeneration through the GLP2–Wnt1 axis in piglets under ELS. Our research contributes to understanding the mechanism of silicon-rich AMW promoting gut epithelial regeneration and provides a new strategy for the alleviation of ELS-induced IEI.

**Supplementary Information:**

The online version contains supplementary material available at 10.1007/s00018-024-05162-x.

## Introduction

It is well-known that irritable bowel syndrome (IBS) is one of the most general intestinal diseases, which affects between 5 and 10% of otherwise healthy individuals at any one point in time, seriously affecting the quality of life and work efficiency [1]. Stress-induced intestinal epithelial injury (IEI) in infancy and a delay in repair are predisposing factors for refractory gut diseases in adulthood [2], such as IBS. Meanwhile, stress, especially early-life stress (ELS), can increase the risk of multiple chronic diseases in adulthood and premature mortality across the lifespan. Notably, in addition to the IBS, ELS exposure-induced intestinal injury and hypothalamus–pituitary–adrenocortical (HPA) axis activation are linked to a higher incidence of anxiety disorders in adults [3, 4]. Therefore, it is necessary to carry out appropriate prevention strategies for ELS.

Maternal separation (MS), also regarded as weaning in mammals, is a familiar ELS paradigm [5–7]. Meanwhile, weaning is a basic process that most mammalian newborns, including humans, must undergo [8, 9]. In rodents, MS is now a valid animal model of IBS because it exhibits the characteristic phenotype of IBS: severe IEI and gut–brain–microbiome (GBM) axis system disorder [2, 6]. Although the phenotype of MS piglets is not completely consistent with that of IBS, there is sufficient evidence that MS stress induces severe IEI and damage repair stagnation in piglets [10, 11]. Of note, the intestinal environment of piglets is quite similar to that of humans, which provides advantages in studying the corresponding disease models [12]. Thus, the MS piglet is a perfect model for investigating and developing innovative prevention and treatment strategies for ELS-induced IEI.

Silicon, apart from oxygen, is the most enrich chemical element in the earth's crust and one of the essential ingredients of life [13]. Considering the contents of silicon in nature and animals, it is expected to exert a vital capacity in animal health. However, the strong physiological effects of silicon seem to be out of proportion to the researchers' attention, and few people have noticed its therapeutic effect. Later, it was determined that silicon is linked to Alzheimer disease [14], cardiovascular protection [15], and immune system augmentation [13], as well as the production of collagen and bone tissue [16], indicating that silicon has a crucial physiological role in animals. According to research, elevating silicon consumption can not only enhance bone growth and development [16], but also boost collagen formation and repair connective tissue injury, implying its potential injury repair efficacy [17]. Previous studies found silicon's immune stimulating activity in pig [18] and horse [19] animal models. Increasingly evidence, either directly or indirectly, proves the anti-inflammatory and antioxidant effects of silicon [20]. The wholesome effects of silicon-rich zeolite or compounds have been reported, such as inhibiting cancer cell proliferation and intestinal inflammatory response [13]. Water is an indispensable necessity in the daily diet and exerts a significant effect on the biochemical and physiological processes of the gut. Moreover, the content of silicon ions is also used to assess the quality of natural alkaline mineral water. Hence, silicon supplementation through drinking water may be an efficient and low-cost approach. However, to our knowledge, the effect of silicon-rich water on ELS-induced IEI and the specific mechanism involved are still not elucidated. In this study, we prepared a silicon-rich alkaline mineral water (AMW, pH = 8.8) using sodium metasilicate pentahydrate (SMP) and explored its effects on IEI induced by ELS (MS/weaning stress) in piglets. Our research demonstrates, for the first time, that silicon-rich AMW has a positive effect on the repair of IEI induced by ELS, providing a new perspective for the prevention and treatment of ELS in young mammals.

## Materials and methods

### Ethics statement

Experiment consent was obtained from the Northeast Agricultural University (NEAU) Animal Ethics Committee (consent ID: NEAUEC202102415). All experimental procedures for lowering pain in piglets were carried out after receiving approval from the Guide for the Care and Use of Laboratory Animals at NEAU, Harbin, China.

### Animal model and experimental design

The model of MS piglets was used according to previous study [5]. A total of 240 piglets from 40 different sows (3 or 4 parity, 6 piglets per litter) were selected. All piglet suddenly separated from sows at 28 days of age, and reared in a strange environment for 15 days. To induce the maximum ELS (MS/weaning stress and social hierarchy stress), animals from the same litter were divided into different replicates. Then, 12 replicates were randomly divided into two drinking water treatment groups (six replicate pens per group and 20 piglets per pen): (1) Control group, consumed basal water (pH = 7.0); and (2) SMP group, consumed silicon-rich AMW (basal water + 500 mg/L SMP). Various studies clearly demonstrated that, compared with un-weaned piglets, MS/weaning stress causes severe IEI in piglets, as manifested by intestinal villus atrophy and functional cell loss [5, 21]; thus, no stress-free control group was set in this study. All piglets were administered an identical feed diet (NRC, 2012) and had unlimited access to food and water in this study. The dosage of SMP added to silicon-rich AMW was based on previous study [5]. Food-grade purity SMP was purchased from Nail Biotechnology Co., Ltd. (Beijing, China).

### Sample collection

On the last day, one piglet was randomly selected from each replicate for sample collection. Serum samples were obtained as previously studies [22]. In brief, blood samples (10 mL each) were collected from the jugular, and the serum was obtained by immediate centrifugation (10 min, 4 ℃, 3000 × g) and kept at − 80 ℃ for analysis. The samples of tissues, paraffin section, scanning electron microscopy (SEM), and transmission electron microscopy (TEM) were collected and stored at corresponding temperatures for various analyses. Mid-jejunum and mid-ileum mucosal samples were separated using a glass slide.

### Gut morphological damage analysis

As previously described, hematoxylin and eosin (HE) staining [5], SEM [9], and TEM [23] were used to examine intestinal morphology damage. The Philips Model SU8010 FASEM (HITACHI, Japan) was used for SEM visualization. The H-7650 (HITACHI, Japan) was employed to observe the ultrastructure of the small intestine (SI). The villous height (VH) and crypt depth (CD) of each segment were assessed with Image J software, and the ratio of VH:CD was calculated. For each group, a minimum of 6 villi from each sample were determined.

### ELISA assay

The collected serum, mucosal samples, or cell cultures were used to measure the levels of principal SI hormones (peptide YY (PYY), 5-hydroxy-tryptamine (5-HT), neurotensin (Nts), glucagon-like peptide (GLP)1, and GLP2) by ELISA kits following the manufacturer’s protocol. Standards and samples were added in triplicate; the absorbance was valued at 450 nm. The details of commercial ELISA kits can be found in the key resources table (Table S1) in the Supplementary materials.

### Immunohistochemistry and immunoblotting

The details of the commercial reagents, antibodies, and kits used in this study can be found in the key resources table (Table S1) in Supplementary materials.

The preparation and processing of frozen sections and immunofluorescence (IF) staining are consistent with previous study [24]. Briefly, the samples were cut into 8 μm thick sections, washed with TBS, blocked with 10% goat serum for 0.5 h, incubated with the primary antibody working solution at 4 ℃ for 12 h, and then incubated with the corresponding secondary antibody at 37 ℃ for 1 h. A Leica fluorescence microscope (DMi8, Leica, Germany) was used for visualization after DAPI staining. For cell IF, the cells were firstly covered with sufficient neutral formaldehyde (4%, prepared with TBS buffer), fixed at 4 °C for 15 min, washed with TBS, and blocked with 5% goat serum, and then incubated with primary antibody and secondary antibody, and finally nucleus stained with DAPI for 10 min. Periodic acid Schiff (PAS) staining of the jejunum and ileum was employed to evaluate the goblet cell differentiation using a commercial kit (Solarbio, Beijing, China) as previously [24]. For Western blot, total proteins of tissues or cells were separated by RIPA Lysis buffer. The detailed procedure for western blot is shown in our previous report [5].

### Quantitative real-time PCR (qRT-PCR)

Total RNA was extracted using an RNAout reagent and reverse-transcribed using the TIANScript RT Kit (TIANGEN, Inc., Beijing, China). The primers used in this study for qRT-PCR (Table S2) were designed by the Primer Premier software 6. The relative mRNA level was calculated using the 2^−ΔΔCT^ method.

### RNA-sequencing (RNA-Seq) analysis and gene set-enrichment analysis (GSEA)

Total RNA of mid-jejunum tissues was extracted and sequenced by Wekemo Tech Group Co., Ltd. (Shenzhen, China). The data was analyzed by HISAT2 v2.0.5, and differentially expressed genes (DEGs) were analyzed by DESeq2 R package 1.16.1 (*P* < 0.05, fold change threshold:1). ClusterProfiler R package (3.4.4) was used to test the statistical enrichment of differential expression genes in KEGG pathways. Various functional and characteristic gene signatures were applied for GSEA using GSEA software (v4.1.0). The statistical significance was determined by comparing the enrichment score and the enrichment results generated from the random arrangement of 1000 genomes to obtain the p value (nominal *p* value). A more detailed “material and methods of RNA-Seq” is provided in the Supplementary materials.

### Cell culture and treatment

The porcine intestinal epithelial cells (IPEC-J2) were obtained from the Prof. Dong Na Lab (NEAU, Harbin, China). IPEC-J2 cells were cultured in DMEM/F12 plus 10% (v/v) fetal bovine serum [24], and all cells were placed in an incubator at 37℃ and 5% CO_2_. The establishment of lipopolysaccharide (LPS)-induced intestinal injury model was based on the results of CCK8 cell viability assay (Dojindo, Kumamoto, Japan). In brief, IPEC-J2 cells were plated in 96-well plates and then treated with LPS at different concentrations (0, 2.5, 5, 10, 20, and 40 μg/mL) for various times (2, 4, 6, 8, and 10 h). After the treatments, 10 μL of CCK-8 assay solution was added to each well, and the cells were further incubated for 1.5 h at 37 °C. The absorbance was then measured at 450 nm. Moreover, we evaluated the optimal concentration of SMP (0.3 mg/mL) through the CCK8 assay. For cell treatment design, firstly, we evaluated the effect of SMP on the proliferation of IPEC-J2 cells under physiological conditions. Subsequently, in vitro, we verified the hypothesis that GLP2/GLP2 receptor (GLP2R)-dependent Wnt1/β-catenin signaling mediated SMP to maintain gut regeneration by promoting cell proliferation and differentiation. The GLP2 and GLP2R antagonist GLP2^3−33^ were bought from MedChemExpress company (New Jersey, USA). The doses of GLP2 (100 nmol/L) and GLP2^3−33^ (100 nM, MCE, New Jersey, USA, EC_50_ = 5.8 nM) given to cells are selected primarily according to previous study and the manufacturer description [25]. The details of cell culture treatment are shown in Figs. 5A and 6A. Briefly, IPEC-J2 or silencing-Wnt1 IPEC-J2 cells were treated with PBS (Con), SMP, GLP2, SMP plus GLP2^3−33^ for 12 h, and then all cells, except the Con treatment, were cultured under LPS exposure for 6 h. After that, various analyses were performed.

### Gene silencing of Wnt1 using small interfering RNA (SiRNA)

To knock down Wnt1 expression, the cells were transiently transfected with SiRNA that targets Wnt1 (SiWnt1); sense strand, 5′-CCGAUUCCAAGAGUCUGCAACUGGU-3′, antisense strand, 5′-ACCAGUUGCAGACUCUUGGAAUCGG-3′, Tsingke Biotechnology Co., Ltd., Beijing, China) and negative control SiRNA (nontargeting SiRNA; sense strand, 5′-CCGCUACGAGACUGUACCAUUAGGU-3′, antisense strand, 5′-ACCUAAUGGUACAGUCUCGUAGCGG-3′) as described previously [26]. IPEC-J2 cells in 6-well plates were transfected with 2 μg of SiWnt1 or control SiRNA using Lipofectamine 2000 (Invitrogen) in Opti-MEM according to the manufacturer's instructions. After 6 h, the cells were refreshed with complete medium for 48 h and then stimulated with SMP and LPS.

### 5-Ethynyl-2′-deoxyuridine (Edu) incorporation assay

Cell propagation was cytochemically detected according to the manufacturer’s instructions (C0071L, Beyotime, China). Briefly, IPEC-J2 cells were incubated with the Edu staining buffer for 2.5 h, fixed with 4% polyformaldehyde, and stained with DAPI. The stained cells were scanned and photographed under a Leica fluorescence microscope (DMi8, Leica, Germany). Furthermore, the Edu^+^ cell ratio was used to assess Edu-positive cells with Image J software (National Institute of Health, USA).

### Cell mitochondrial membrane potential assay by JC-1 staining

The decreased mitochondrial membrane potential is a hallmark of early apoptosis. Following the manufacturer’s instructions (C2003S, Beyotime, China), IPEC-J2 cells were stained with JC-1 for 20 min at 37 ℃. A DMi8 Leica fluorescence microscope was used for visualization of JC-1 staining. Red fluorescence (high potential) indicated JC-1 aggregates in the matrix of mitochondria to form polymers (J-aggregates); green fluorescence represented that JC-1 was concentrated in the cytoplasm, and JC-1 was a monomer (low potential).

### Flow cytometry for cell cycle

The cell cycle process was determined by flow cytometry, and the procedure was consistent with previously [27]. Briefly, IPEC-J2 cells were inoculated into a 6-well plate. After the predetermined treatment procedure, the cells were digested by trypsin and collected into 1.5 mL centrifuge tubes. Subsequently, the cells were fixed with 70% ethanol at 4 ℃ for 12 h and then resuspended with PBS to wash the cells, followed by propidium staining at 37 ℃ and incubation in the dark for 30 min. Finally, we subjected them to flow cytometry at an excitation wavelength of 488 nm. The results of the cell cycle were analyzed using ModiFIT software.

### Wound-healing assay

Wound-healing assay was used to evaluate the injury repair potential of SMP as described previously [28]. Cells were seeded on 6-well plates at a density of 1 × 10^6^ cells/well. After being confluent, cells were differentiated and polarized for 5 days in the culture medium. Then, cells were subjected to various treatments according to the predetermined design. Subsequently, the cells were uniformly scratched with a 300 μL tip at the same time. The scratch images at 0, 4, 8, 12, and 24 h after treatment were recorded. The wound healing rate was calculated by Image J software.

### Transepithelial electrical resistance (TER) measurement

TER was detected as previously described [29]. Briefly, cells were inoculated into a 6-well plate (set as a blank control without cells) that featured a transwell chamber with 4.5-m pores (Costar, Coring Inc., New York, NY, USA). Cells were differentiated and polarized for 5–7 days in the culture medium. Then, the cells were treated with different reagents. After the predetermined treatment procedure, Millicell ERS-2 (EMD Millipore, Billerica, MA) was employed to read the TER value.

### Statistical analysis

All data were analyzed by STAMP and GraphPad Prism 9.0 (GraphPad Software, San Diego, CA, USA) software. Statistical analysis was calculated using Student’s *t* tests to compare differences between the two groups or one-way ANOVA for multiple group comparison, followed by Tukey’s post hoc pairwise comparison. Difference significance was set to 0.05 (*P* < 0.05), and all error deviations are described by ± SD. The specific descriptions of the statistical means are shown in the figure legends.

## Results

### Silicon-rich AMW repaired ELS-induced IEI

**Fig. 1:**
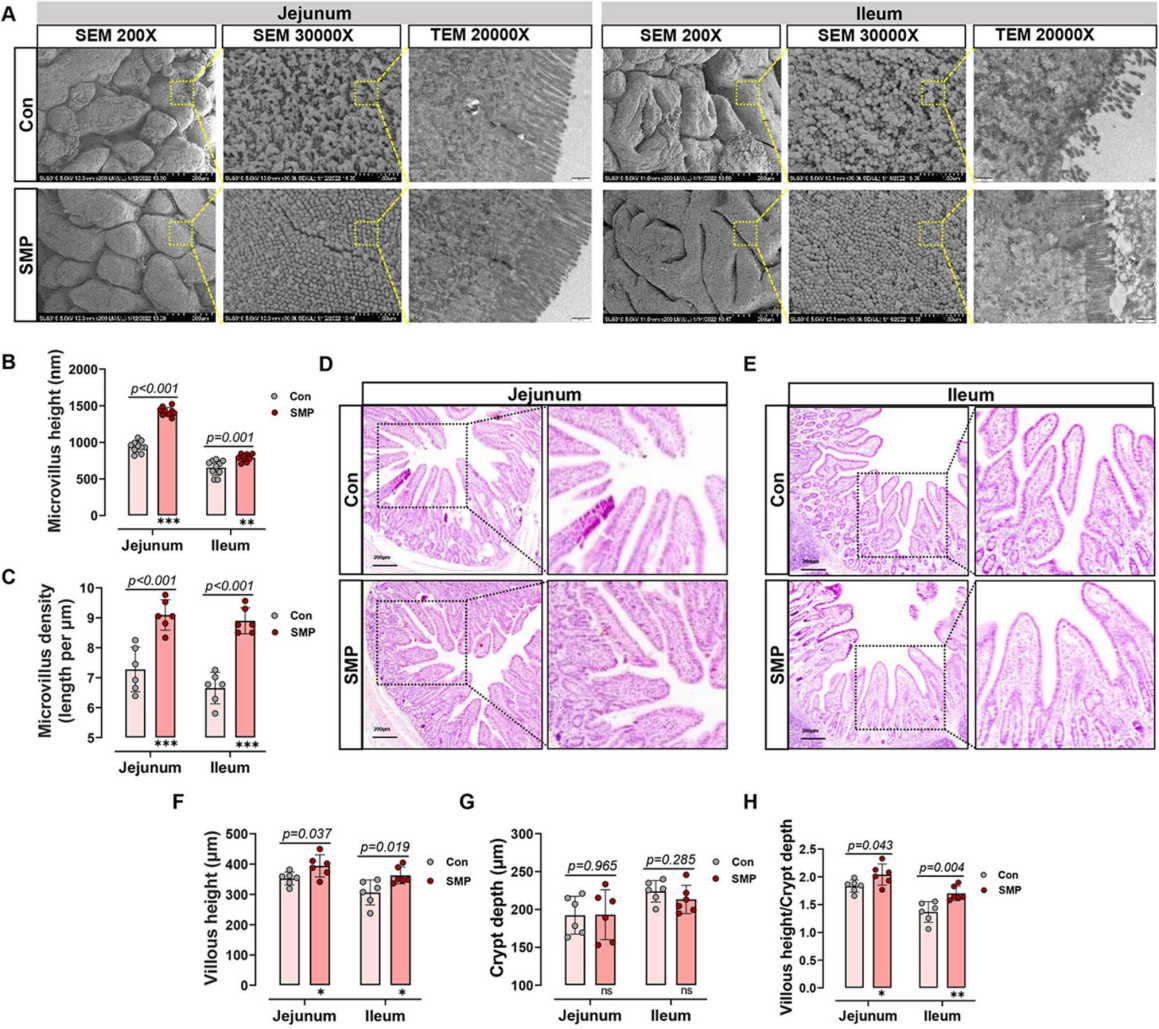
Silicon-rich alkaline mineral water repaired stress-induced intestinal injury in MS piglets. **A** Morphological observation of the small intestine by SEM (200X and 30000X) and TEM (20000X). **B** Microvillus height and **C** microvillus density statistical analysis. **D** Jejunum and **E** ileum tissue morphological observation by H&E staining. **F** Villous height, **G** crypt depth, and **H** villous height/crypt depth ratio statistical analysis. Data are presented as the mean ± SD. Statistical analysis was performed using Student’s *t* tests to compare differences between the two groups. ^ns^*P* > 0.05, *P < 0.05, **P < 0.01, and ***P < 0.001

Gut epithelial morphology and microvilli characteristics are the common indicator for judging intestinal health [10]. In this study, MS stress-induced significant epithelial damage, but this damage could be repaired by silicon-rich AMW (Fig. 1A), as evidenced by microvilli height (Fig. 1A, B) and density (Fig. 1A, C) in the jejunum and ileum. Moreover, HE staining demonstrated that SMP improved the SI morphology (Fig. 1D, E), with an elevated VH and VH: CD ratio (Fig. 1F, G). These data indicate that silicon-rich AMW repairs damaged intestinal epithelium induced by MS stress in piglets.

### Silicon-rich AMW activated the intestinal GLP2/GLP2R pathway in MS piglet

**Fig. 2:**
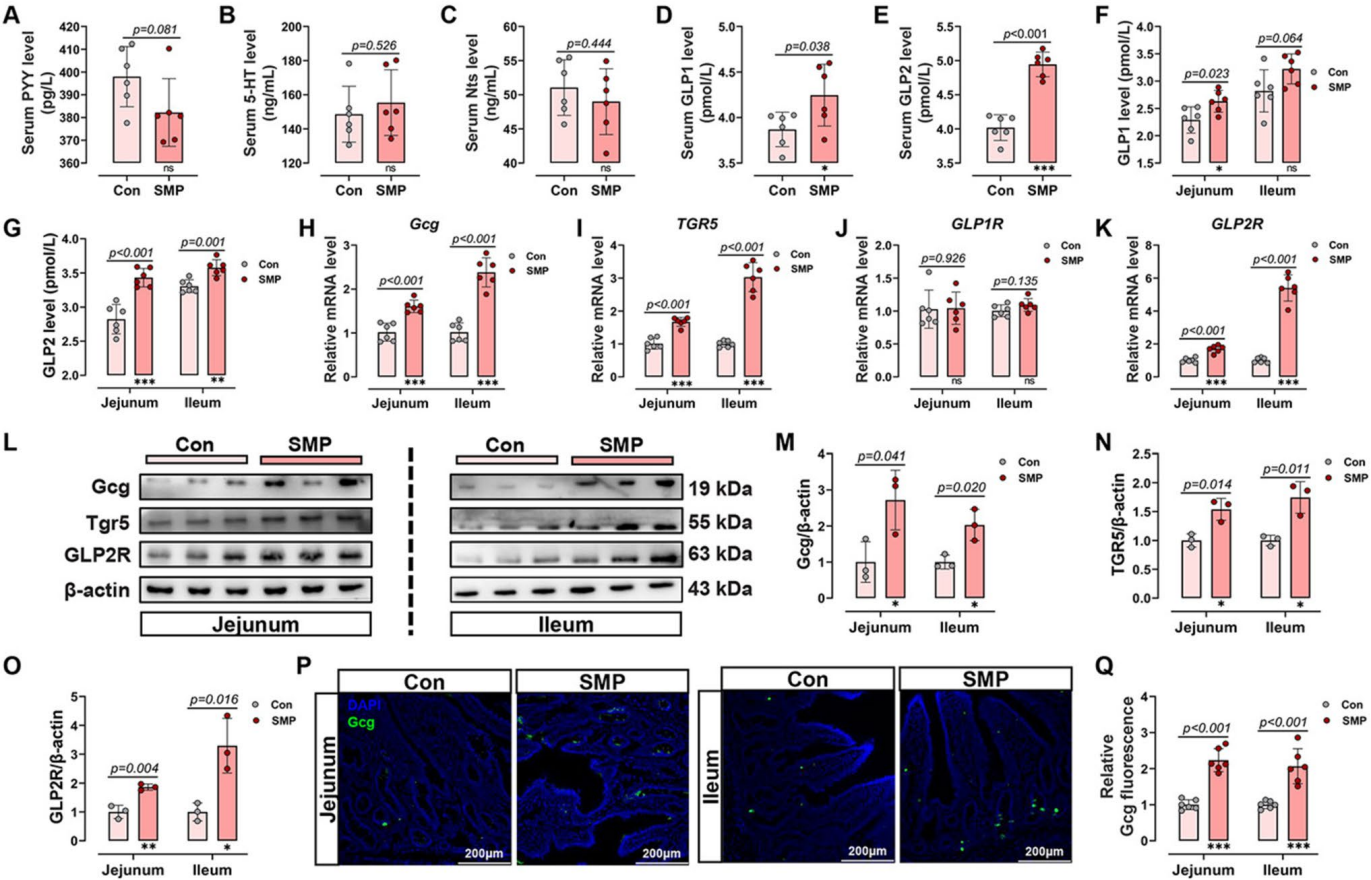
Silicon-rich alkaline mineral water activated the intestinal GLP2/GLP2R pathway in MS piglet. **A** PYY, **B** 5-HT, **C** Nts, **D** GLP1 and **E** GLP2 level in the serum. **F** GLP1 and **G** GLP2 level in the small intestine. **H** Gcg, **I** TGR5, **J** GLP1R and **K** GLP2R mRNA expression level in the small intestine. **L** Western blot analysis of Gcg, TGR5, and GLP2R protein expression in the small intestine. **M**–**O** Relative protein levels of **M** Gcg, **N** TGR5, and **O** GLP2R in the small intestine. **P** Immunofluorescence images of Gcg staining (green) and DAPI (blue) in the small intestine. **Q** Relative Gcg fluorescence. Data are presented as the mean ± SD. Statistical analysis was performed using Student’s *t* tests to compare differences between the two groups. ^ns^*P* > 0.05, *P < 0.05, **P < 0.01, and ***P < 0.001

Gut-derived hormones are regulated by numerous systems and are essential for maintaining intestinal homeostasis [30]. We next analyzed the main SI hormones, including PYY, 5-HT, Nts, GLP1, and GLP2 (Fig. 2A–E). SMP notably improved the serum levels of GLP1 and GLP2 (Fig. 2D, E), but had no impact on others (Fig. 2A–C). Subsequently, both hormone content in SI mucosa were detected. Results showed that the jejunum levels of GLP1 (Fig. 2F), the jejunum and ileum levels of GLP2 (Fig. 2G) were increased by SMP treatment. Meanwhile, the expression levels of glucagon (Gcg, encoding GLP1 and GLP2, Fig. 2H, L, M), TGR5 (the GLP secretion trigger, Fig. 2I, L and N), and GLP2R (Fig. 2K, L and O) were increased in the SI. However, there was no apparently alternation in the mRNA expression of GLP1R between the two groups (Fig. 2J). In addition, the Gcg^+^ cell numbers of SI were also significantly increased in SMP group piglets (Fig. 2P, Q). Both GLP1 and GLP2 are secreted by endocrine cells in the SI. Among them, GLP2 has been demonstrated to increase nutrition absorption and IEI repair in a swine model, while GLP1 plays a role in glucose metabolism homeostasis [31]. In summary, given the physiological functional properties of both hormones and our results, it is suggested that the GLP2/GLP2R pathway is involved in the silicon-rich AMW repair of damaged intestinal epithelium.

### Silicon-rich AMW facilitated Lgr5^+^ ISC proliferation by targeting the G1–S phase cell cycle checkpoint via stimulating the Wnt1/β-catenin pathway

RNA-Seq was utilized to explore the mechanism by which silicon-rich AMW mitigated MS stress-induced IEI. KEGG-enrichment analysis showed the enrichment of various pathways, including the “Wnt signaling pathway”, “cell cycle,” and “DNA replication” (Fig. S1A). Meanwhile, the pathways of “embryonic stem cell core”, “cell cycle mitotic”, “cell cycle checkpoints”, “G1_S cell cycle”, “targets of ccnd1 (Cyclin D) and cdk4” were upregulated (Fig. S1B–F), and the pathway of “inflammatory response and cholesterol up” was down-regulated (Fig. S1G) in SMP treatment piglet’s jejunum according to GSEA results. Therefore, the effects of SMP on Wnt signaling, intestinal cell proliferation, and cell cycle transition were determined in MS piglets SI to verify the results of RNA-Seq.

**Fig. 3:**
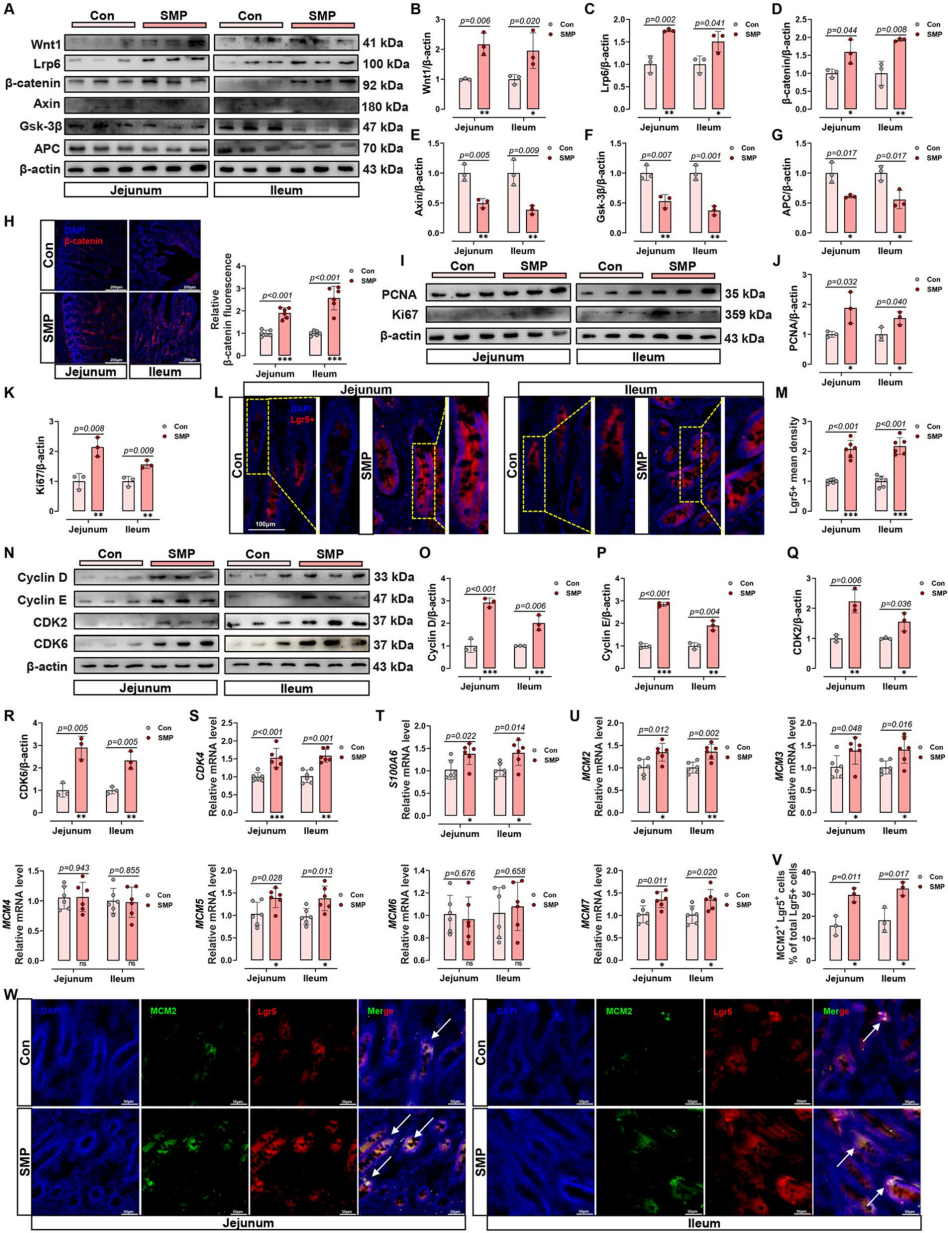
Silicon-rich alkaline mineral water facilitated Lgr5^+^ ISC proliferation by targeting the G1–S phase cell cycle checkpoint via stimulating the Wnt1/β-catenin pathway. **A** Western blot analysis of Wnt1/β-catenin signaling pathway activity. **B**–**G** Relative protein levels of **B** Wnt1, **C** Lrp6, **D** β-catenin, **E** Axin, **F** GSK-3β, and **G** APC in the small intestine. **H** Immunofluorescence images and fluorescence statistical analysis of β-catenin. **I** Western blot analysis of PCNA and Ki67 protein expression. **J**, **K** Relative protein levels of **J** PCNA and **K** Ki67 in the small intestine. **L** Immunofluorescence images of Lgr5^+^ cell staining (Red) and DAPI (blue). **M** Statistical analysis of Lgr5^+^ mean density. **N** Western blot analysis of G1/S-phase transition-related protein expression in the small intestine. **O**–**R** Relative protein levels of **O** Cyclin D, **P** Cyclin E, **Q** CDK2 and **R** CDK6 in the small intestine of MS piglet. **S**–**U** Relative mRNA levels of **S** CDK4, **T** S100A6, and **U** MCM 2–7 in the small intestine. **V** Statistical analysis of MCM2^+^Lgr5^+^ cells % of total Lgr5^+^ cell. **W** Immunofluorescence images of MCM2^+^ cell staining (green), Lgr5^+^ cell staining (Red) and DAPI (blue) of the small intestine. Data are presented as the mean ± SD. Statistical analysis was performed using Student’s *t* tests to compare differences between the two groups. ^ns^*P* > 0.05, *P < 0.05, **P < 0.01, and ***P < 0.001

Wnt signaling controls several biological pathways, including ISC renewal, cell proliferation, and differentiation [32]. Based on the results of RNA-Seq, we speculated that silicon-rich AMW promotes ISC proliferation by activating Wnt signaling to mediate cell cycle passage through the G1/S-phase checkpoint, accordingly maintaining gut epithelial regeneration. The canonical Wnt/β-catenin pathway is activated by Wnt1, which transmits signals through transmembrane receptors, such as Lrp6. Wnt1, acting as a positive regulator by suppressing β-catenin degradation, controls the downstream target genes expression [33]. In our study, SMP upregulated the protein expression of Wnt1, Lrp6, and β-catenin, while reduced β-catenin inhibitors expression, including Axin, GSK-3β and adenomatous polyposis coli (APC, Fig. 3A–G). The increased fluorescence density of β-catenin in the SI after SMP treatment also proved the above results (Fig. 3H). Furthermore, SMP promoted the proliferation of intestinal epithelial cells (IEC), as evidenced by the upregulation of proliferating cell nuclear antigen (PCNA) and Ki67 (Fig. 3I–K). Surprisingly, SMP apparently increased the number of Lgr5^+^ (leucine-rich repeat-containing G-protein-coupled receptor 5) ISC in the SI crypt (Fig. 3L, M), which are regarded as cycling and long-lived multipotent stem cells [34].

**Fig. 4:**
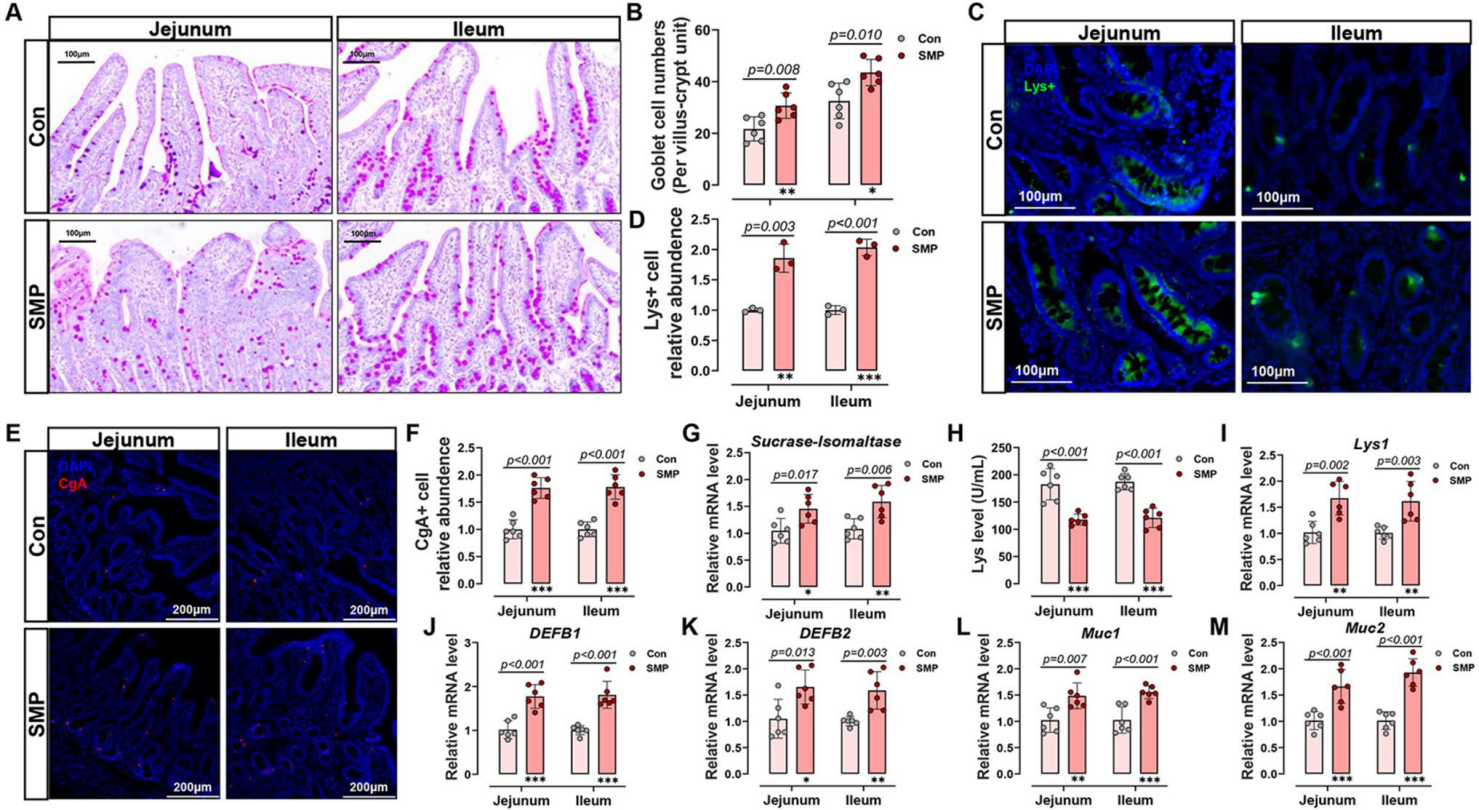
Silicon-rich alkaline mineral water-induced Lgr5^+^ ISC differentiation to maintain intestinal epithelium regeneration. **A** Intestinal goblet cells were shown by PAS staining. **B** Statistical analysis of goblet cell number. **C** Immunofluorescence images of Lys^+^ cell staining (green) and DAPI (blue). **D** statistical analysis of the Paneth cell marker Lys^+^. **E**–**F** Immunofluorescence images and statistical analysis of endocrine cell marker CgA^+^ staining (Red) and DAPI (blue). **G** Statistical analysis of absorptive enterocyte marker sucrase–isomaltase mRNA level. **H** Mucosal lysozyme level in the small intestine. **I**–**K** mRNA levels of Paneth cells related-factors **I** Lyz1, **J** DEFB1 and **K** DEFB2. **L**–**M** mRNA levels of goblet cell related-factors **L** Muc1 and **M** Muc2. Data are presented as the mean ± SD. Statistical analysis was performed using Student’s *t* tests to compare differences between the two groups. ^ns^*P* > 0.05, *P < 0.05, **P < 0.01, and ***P < 0.001

Given that KEGG-enrichment analysis and GSEA results suggested that SMP upregulated the pathways of “DNA replication”, and “G1_S cell cycle” in the SI, we conducted a series of verifications. The G1 (Gap 1), S (DNA synthesis), G2 (Gap 2), and M (mitosis) phases comprise the normal cell cycle in eukaryotes. The initiation of DNA replication means the transition from G1 to S phase, and efficient S phase entry is necessary for tissue development and damage repair [35]. In this study, SMP treatment significantly improved the expression of Cyclin D, Cyclin E, Cyclin-dependent kinase (CDK) 2, CDK 6, CDK4, and S100A6 in the SI of MS piglets (Fig. 3N–T), all of which mediated cell-cycle passage through the G1/S-phase checkpoint [34]. On the other hand, ISC admission to S-phase is mediated, to some extent, through Mini chromosome maintenance (MCM) 2–7 complexes loading onto the DNA origin of replication [34]. Quantitative RT-PCR results indicated that drinking silicon-rich AMW promoted the mRNA levels of MCM2, MCM3, MCM5, and MCM7, whereas not MCM4 or MCM6 (Fig. 3U). Subsequently, to confirm whether an alteration in intestinal G1–S cell cycle checkpoint gene expression existed at the ISC level, multiple staining was employed to assess the colocalization of MCM2 in the SI Lgr5^+^ cells. The results showed that SMP treatment notably elevated MCM2 expression in the Lgr5^+^ ISC (Fig. 4V, W). These data confirm the results of RNA-Seq: silicon-rich AMW promotes the activity of Wnt1/β-catenin pathway to facilitate cell cycle passage through the G1/S-phase checkpoint, accordingly promoting the Lgr5^+^ ISC proliferation and IEC regeneration.

### Silicon-rich AMW-induced Lgr5^+^ ISC differentiation to maintain intestinal epithelium regeneration

The ISC differentiated into the specialized IEC types, including goblet cells (GC, PAS^+^), Paneth cells (PC, Lysozyme, Lys^+^), enteroendocrine cells (Chromogranin A, CgA^+^), and absorptive enterocytes (Sucrase–Isomaltase^+^), to replace damaged cells, which is the basis of gut epithelial regeneration and IEI repair [28]. Interestingly, these specialized IEC markers in the SI of SMP treatment piglets were significantly higher than that in the Con group (Fig. 4A–G), further demonstrating that Wnt1/β-catenin signaling activation drives ISC proliferation and differentiation. Meanwhile, the Lys content, which is secreted by PC, was increased, as well as the mRNA expression of Lys1, beta-defensin (DEFB)1, and DEFB2 in the SI after drinking SMP water (Fig. 4H–K). Similarity, the gene expression of mucin (Muc), Muc1, and Muc2 (secreted by GC) also increased after SMP treatment (Fig. 4L, M). These results indicate that silicon-rich AMW facilitates ISC proliferation and differentiation by activating Wnt1 signaling to mediate cell cycle passage through the G1/S-phase checkpoint, accordingly maintaining gut epithelial regeneration and IEI repair.

### Inhibition of GLP2/GLP2R signaling by GLP2^3−33^ disrupted SMP-induced IPEC-J2 cell regeneration

Since intestinal cell lines that endogenously produce functional GLP2R are few, we used IPEC-J2 cells to verify the SMP's positive effects on the gut in vitro. The IPEC-J2 cell line was initially isolated from the jejunum of newborn piglets and is composed of undifferentiated porcine intestinal crypt-based columnar cells [36]. According to previous studies, it can be confirmed that IPEC-J2 contains GLP2/GLP2R signal transduction [36–38], and importantly, Lgr5^+^ cells and other specialized IEC markers can be observed [39]. Based on the cell viability assay, the optimal concentration of SMP for IPEC-J2 cells was 0.3 mg/mL for 12 h (Fig. S2A), and the LPS-induced intestinal injury model treatment was set at 10 μg/mL LPS exposure for 6 h (Fig. S2B, C). Under physiological conditions, SMP could activate the Wnt1/β-catenin pathway and IPEC-J2 cell proliferation by improving the protein levels of Wnt1, β-catenin, Ki67, and PCNA, as well as Edu^+^ cell ratio (Fig. S2D–I), which was also further verified by the wound healing rate of scratches (Fig. S2J). More importantly, we found the GLP2 level in the supernatant of IPEC-j2 cells was elevated after SMP treatment; meanwhile, it was reduced after LPS exposure, while SMP can restore it to normal (Fig. S2K). These data indicate that the physiological effects of SMP are related to the GLP2-dependent pathway.

**Fig. 5:**
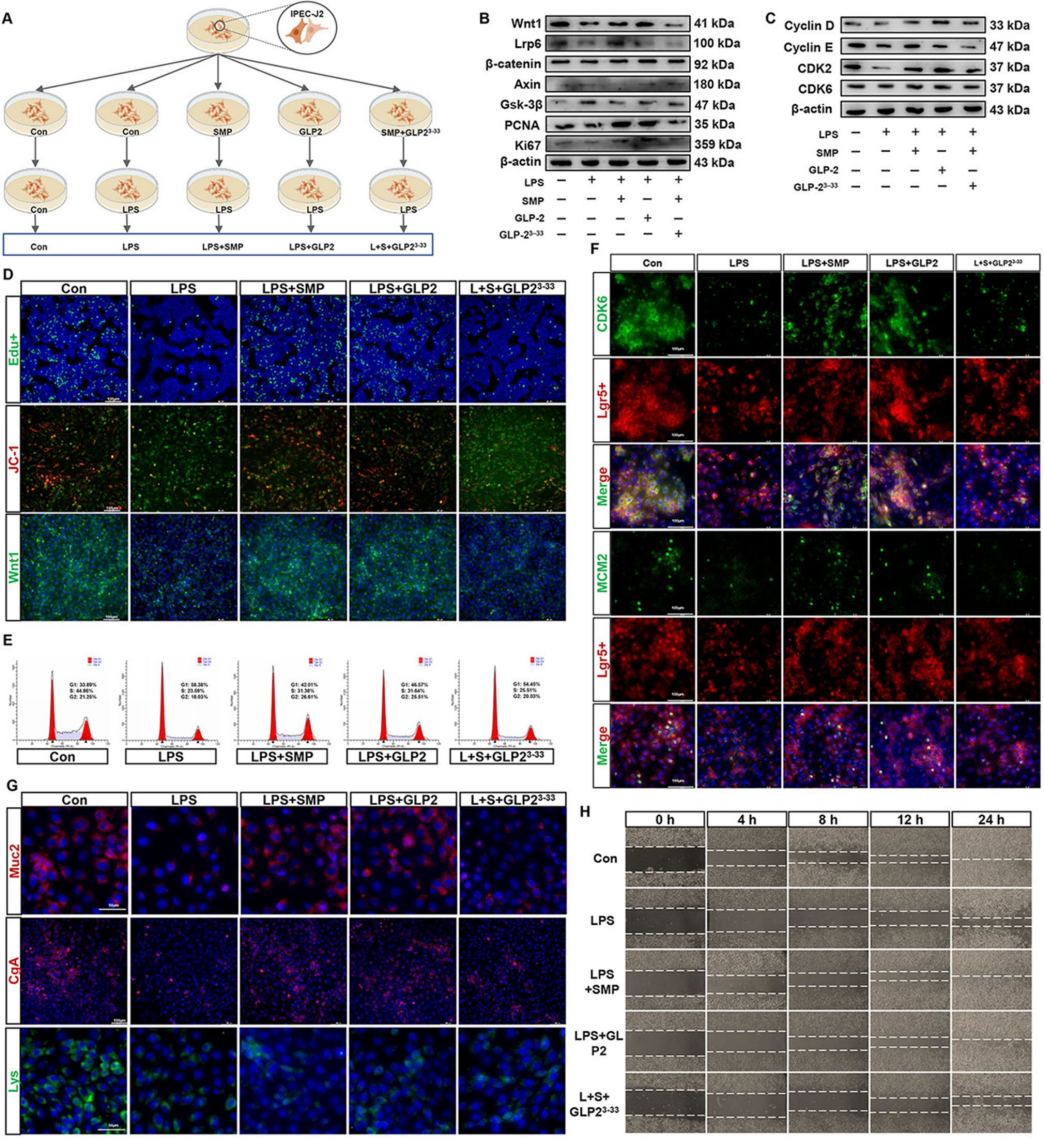
Inhibition of GLP2/GLP2R signaling by GLP2^3−33^ disrupted the regeneration effect of SMP in LPS-challenged IPEC-J2 cells. **A** IPEC-J2 cells were incubated with Con, LPS, SMP + LPS, GLP2 + LPS, and SMP + GLP2^3−33^ + LPS. **B** Western blot analysis of Wnt1/β-catenin signaling pathway activity and proliferation-related proteins in LPS-challenged IPEC-J2 cells. **C** Western blot analysis of G1/S-phase transition-related protein expression. **D** Immunofluorescence images of Edu^+^ (green), JC-1 (red), and Wnt1 (green) staining in LPS-challenged IPEC-J2 cells. **E** Flow cytometry analysis of cell cycle. **F** Immunofluorescence images of CDK6^+^Lgr5^+^ cell and MCM2^+^Lgr5^+^ cell staining. **G** Immunofluorescence images of Muc2 (red), CgA (red), and Lys (green) staining. **H** Wound healing rate in LPS-challenged IPEC-J2 cells

Subsequently, we used the GLP2R inhibitor GLP2^3−33^ to block GLP2/GLP2R signaling (Fig. 5A). In the LPS-induced intestinal injury model, both SMP and GLP2 treatment promoted cell proliferation by activating Wnt1/β-catenin signaling (Figs. 5B, S3A–G). The results of Edu^+^ cell rate, JC-1 polymer/monomer ratio (mitochondrial membrane potential marker), and Wnt1 mean density (Figs. 5D, S3I–K) also supported this. Notably, the GLP2R antagonist GLP2^3−33^ blocked SMP-induced Wnt1/β-catenin signaling activation and its mediated cell proliferation in LPS-challenged IPEC-J2 cells. Moreover, the levels of G1/S-phase transition-related proteins, Cyclin D, Cyclin E, CDK2, and CDK6, were inhibited after LPS exposure, whereas SMP and GLP2 could restore them to normal (Figs. 5C, S3H). Surprisingly, the GLP2^3−33^ administration made this effect of SMP disappear. In addition, flow cytometry for cell cycle analysis further demonstrated that LPS-exposure-induced G1-phase arrest, while SMP and GLP2 interventions stimulated more S-phase entry of IPEC-J2 cells; likewise, GLP2^3−33^ discouraged the promotion effect of SMP by inhibiting the GLP2R signaling (Figs. 5E, S3L). Notably, SMP may promote ISC cell differentiation by promoting Lgr5^+^ cell passage through the G1/S-phase checkpoint via the GLP2-dependent mechanism, as evidenced by the results of CDK6^+^Lgr5^+^ cell ratio and MCM2^+^Lgr5^+^ cell ratio (Figs. 5F, S3M). As expected, the ISC S-phase entry markers, MCM2, 3, 5, and 7 mRNA levels were downregulated by LPS and GLP2^3−33^, whereas they were recovered by SMP and GLP2 (Fig. S3N). Subsequently, to investigate the mechanism of SMP promoting Lgr5^+^ cell differentiation, we examined the cell density of specialized intestinal cell markers, including Lgr5^+^, Lys^+^, Muc2^+^, CgA^+^, and sucrase–isomaltase^+^. SMP and GLP2 did not improve the density of Lys^+^ cells (Figs. 5G, S3O) inhibited by LPS in vitro, suggesting that the differentiation of PC was related to the gut microflora. Of note, SMP and GLP2 had the same efficacy, increasing the cell density of Muc2^+^ and CgA^+^, and the expression of sucrase isomaltase (Figs. 5G, S3P–R) compared to LPS-treated IPEC-J2, while GLP2^3−33^ interrupted the differentiation-promoting effect of SMP. Importantly, the TER results (Fig. S3S) and wound healing rate (Figs. 5H, S3T) further demonstrated that SMP maintained IPEC-J2 regeneration through the GLP2-dependent pathway. These results confirmed the conclusion of animal experiments: SMP water maintains intestinal epithelial regeneration and IEI repair through the GLP2/GLP2R-dependent pathway.

### Silencing Wnt1 by SiWnt1-blunted SMP-stimulated IPEC-J2 cell regeneration

**Fig. 6:**
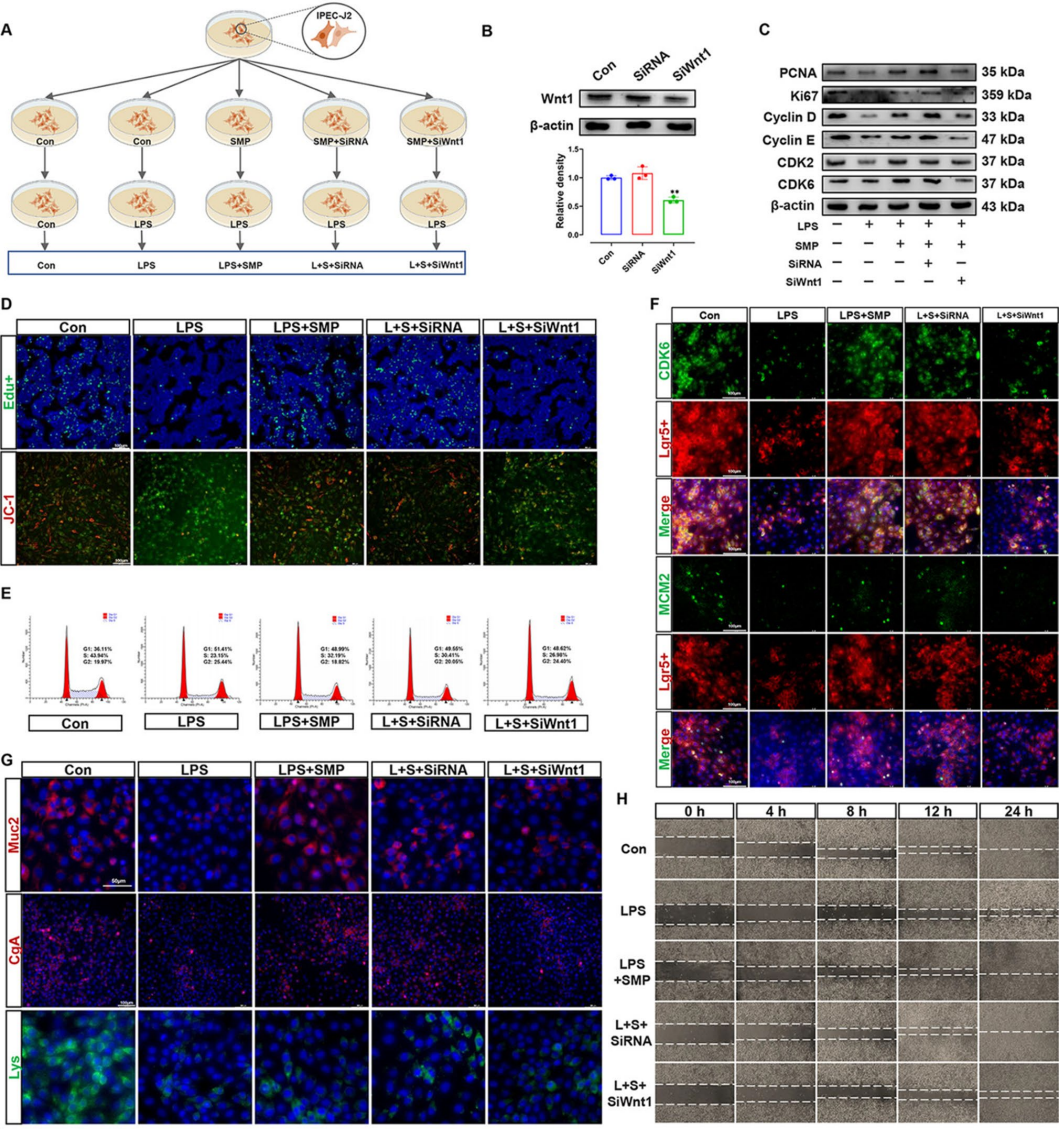
Silencing Wnt1 by SiRNA blunted SMP-stimulated IPEC-J2 cell regeneration. **A** IPEC-J2 cells were incubated with Con, LPS, SMP + LPS, SMP + SiRNA + LPS, and SMP + SiWnt1 + LPS. **B** Protein level of Wnt1 in IPEC-J2 cells 48 h after SiRNA and SiWnt transfection. **C** Western blot analysis of proliferation-related proteins and G1/S-phase transition-related protein expression. **D** Immunofluorescence images of Edu^+^ (green) and JC-1 (red) staining. **E** Flow cytometry analysis of cell cycle. **F** Immunofluorescence images of CDK6^+^Lgr5^+^ cell and MCM2^+^Lgr5^+^ cell staining. **G** Immunofluorescence images of Muc2 (red), CgA (red), and Lys (green) staining. **H** Wound healing rate in LPS-challenged IPEC-J2 cell after silencing Wnt1. Data are presented as the mean ± SD. Statistical analysis was calculated using one-way ANOVA for multiple group comparison followed by Tukey’s post hoc pairwise comparison. ^ns^*P* > 0.05, *P < 0.05, **P < 0.01, and ***P < 0.001 vs. the Con group

To investigate whether the IEI repair effect of SMP depends on Wnt1 signaling, SiRNA was employed to mediate the silencing of the gene encoding Wnt1 (Fig. 6A). After SiWnt1 transfection, the protein expression of Wnt1 in IPEC-J2 cells was inhibited significantly (Fig. 6B). This indicated that the SiWnt1 used in this study was an effective SiRNA for silencing endogenous Wnt1 gene expression. As shown in Fig. 6C, D, silencing Wnt1 inhibited SMP-induced recovery of cell proliferation (Fig. S4A, B, G, H). Meanwhile, the effect of SMP on promoting the cell cycle of LPS-exposed IPEC-J2 cells to passage through the G1–S-phase checkpoint was also blocked by SiWnt1 (Figs. 6C, S4C–F). In addition, flow cytometry for cell cycle analysis further indicated that SMP did not rescue the G1-phase arrest and S-phase entry barrier induced by LPS in the IPEC-J2 cells after silencing Wnt1 (Figs. 6E, S4I). Likewise, the ISC S-phase entry markers CDK6^+^Lgr5^+^ cells and MCM2^+^Lgr5^+^ cells (Figs. 6F, S4J) and MCM2, 3, 5, and 7 (Fig. S4K) were downregulated after silencing Wnt1 in SMP and LPS cocultured IPEC-J2 cells. Of note, like GLP2^3−33^, silencing Wnt1 by SiWnt1 disrupted the differentiation-stimulating effect of SMP (Figs. 6G, S4L–O). On the other hand, TER (Fig. S4P) and scratch healing rate results (Figs. 6H, S4Q) further verified that SMP maintained IPEC-J2 cell regeneration through Wnt1 signaling. Therefore, these results further demonstrate that SMP maintains IEC regeneration and IEI repair by stimulating cell cycle passage through the G1–S-phase checkpoint to provoke cell proliferation and Lgr5^+^ ISC differentiation via activating the GLP2-dependent Wnt1/β-catenin signaling pathway.

## Discussion

The MS piglet model displays pre- and onset IBS phenotypes, including loss of gut barrier integrity, recurrent diarrhea, an active HPA axis, and dysbiosis [6, 10]. Our previous study has proven that SMP water confers MS piglet diarrhea resistance by relieving the duodenum inflammatory response and enhancing tight junction and adherens junction functions [5]. However, the specific molecular mechanism of silicon-rich AMW to improve gut homeostasis is still a mystery. In this study, we demonstrate that drinking silicon-rich AMW maintains intestinal epithelium regeneration through the GLP2–Wnt1 axis in piglets under ELS.

IEI is the direct cause of intestinal dysfunction, which not only facilitates the spread of pathogenic bacteria and inflammation but also weakens the nutrients absorption [10, 40]. Finger-like villi and microvilli projecting from the absorptive enterocytes constitute the SI prominent structure and intact mechanical barrier. Through morphological observation using SEM, TEM, and HE staining, it can be confirmed that MS stress causes severe damage to the intestinal epithelium of piglets. Surprisingly, weaned piglets drinking silicon-rich AMW exhibited more complete epithelial morphology, a higher VH and VH/CD ratio, as well as higher microvilli density and height. This suggests that silicon-rich AMW has the ability to repair damaged intestinal epithelium and villi atrophy.

SI responds rapidly to diet-related stimuli by releasing hormones that can exert powerful regulatory effects with small doses to maintain intestinal homeostasis [30]. Therefore, we evaluated the serum levels of main hormones derived from SI and found that the levels of GLP1 and GLP2 were dramatically elevated after SMP treatment. As an enteroendocrine tissue hormone, both GLP1 and GLP2 are released by gut L-cells. A study on acute graft-versus-host disease showed that GLP2 restores gut homeostasis by promoting the regeneration of ISC and PC, as well as regulating the gut microbiome [41]. In addition, unlike GLP2, the physiological function of GLP1 is primarily to regulate glucose metabolism, and it is considered a glucose concentration-dependent hypoglycemic hormone [42]. Obviously, the phenotypes observed in this study correspond to the potential role of GLP2. In addition, the increased expression of Gcg, TGR5, and GLP2R also demonstrated that silicon-rich AMW activates GLP2/GLP2R signaling in the SI of MS piglets, and this pathway may mediate silicon-rich AMW to improve IEI and epithelial regeneration.

Based on KEGG-enrichment analysis and GSEA by the RNA-seq, the enrichment pathways in the current study constitute a complete and long chain of signaling that is related to intestinal regenerative phenotype [28, 34, 39]. That led us to make a reasonable speculation and to validate it accordingly. We speculated and verified that silicon-rich AMW promotes the proliferation and differentiation of Lgr5^+^ ISC by activating Wnt1/β-catenin signaling to mediate cell cycle passage through the G1/S-phase checkpoint, accordingly maintaining gut epithelial regeneration and IEI repair. In mammals, the IEC is updated every 3–5 days [34]. Continuous regeneration occurs through the rapid proliferation and differentiation of ISC, predominantly Lgr5^+^ ISC, to committed progenitor cells, and then to specific IEC types [43]. When ISC niche equilibrium is disturbed, gut self-healing and barrier integrity suffer. The proliferation and differentiation of crypt stem cells are tightly regulated by signaling networks, and the main driving force is Wnt1/β-catenin signaling [44]. For ISC, most of them are in the unlicensed G1-phase; this extended unlicensed G1-phase allows the ISC to respond to surrounding niche signals, including Wnt signaling and its activator, R-spondin [34]. Passage of the G1–S checkpoint means ISC entry into the DNA synthesis phase of the S phase, the initiation point of cell mitosis [35]. In our study, we found that silicon-rich AMW activated Wnt1/β-catenin signaling, increased the expression of proliferation-related proteins and S-phase entry-related proteins, and elevated the number of Lgr5^+^ cells and specialized IEC, including GC, PC, endocrine cells, and absorptive enterocytes. Therefore, these results prove the original hypothesis.

In a study with mice consistent with this study, GLP2 was discovered to strongly accelerate ISC cell cycle entry into S-phase in a GLP2R-dependent manner [34]. Moreover, earlier research has shown that GLP2 is a powerful activator of gut proliferation as well as Msi-1 transcription in the crypt [45]. These reports suggest that GLP2 is an extrinsic regulator of Wnt-dependent ISC behavior. Although several gut growth factors have been recorded in the studies, the potential interaction of GLP2 with intestinal Wnt signaling and the specific molecular mechanism that promotes ISC proliferation and differentiation have not been thoroughly investigated. In this study, LPS exposure reduced GLP2 expression in IPEC-J2 cells, whereas SMP treatment could restore it. Meanwhile, inhibition of the GLP2/GLP2R pathway by GLP2^3−33^ disrupted the ameliorative effect of SMP in LPS-challenged IPEC-J2 cells, including Wnt1/β-catenin signaling activation-mediated IEC proliferation, stimulating S-phase entry and differentiation of Lgr5^+^ cells, as well as promoting wound healing. Of note, silencing Wnt1 by SiRNA also blunted SMP-stimulated regeneration by inhibiting cell cycle passage through the G1/S-phase checkpoint, and Lgr5^+^ cells differentiated into specialized IEC in LPS-exposure IPEC-J2 cells. Surprisingly, after silencing Wnt1 or inhibiting GLP2/GLP2R signaling in vitro, SMP could not reverse the decreased Lys^+^ cell ratio induced by LPS, suggesting that gut microbiota plays a key role in PC renewal. Apparently, these data indicate that the GLP2/GLP2R pathway and its downstream Wnt1/β-catenin pathway are targets of the intestinal epithelium regeneration mediated by SMP.

**Fig. 7:**
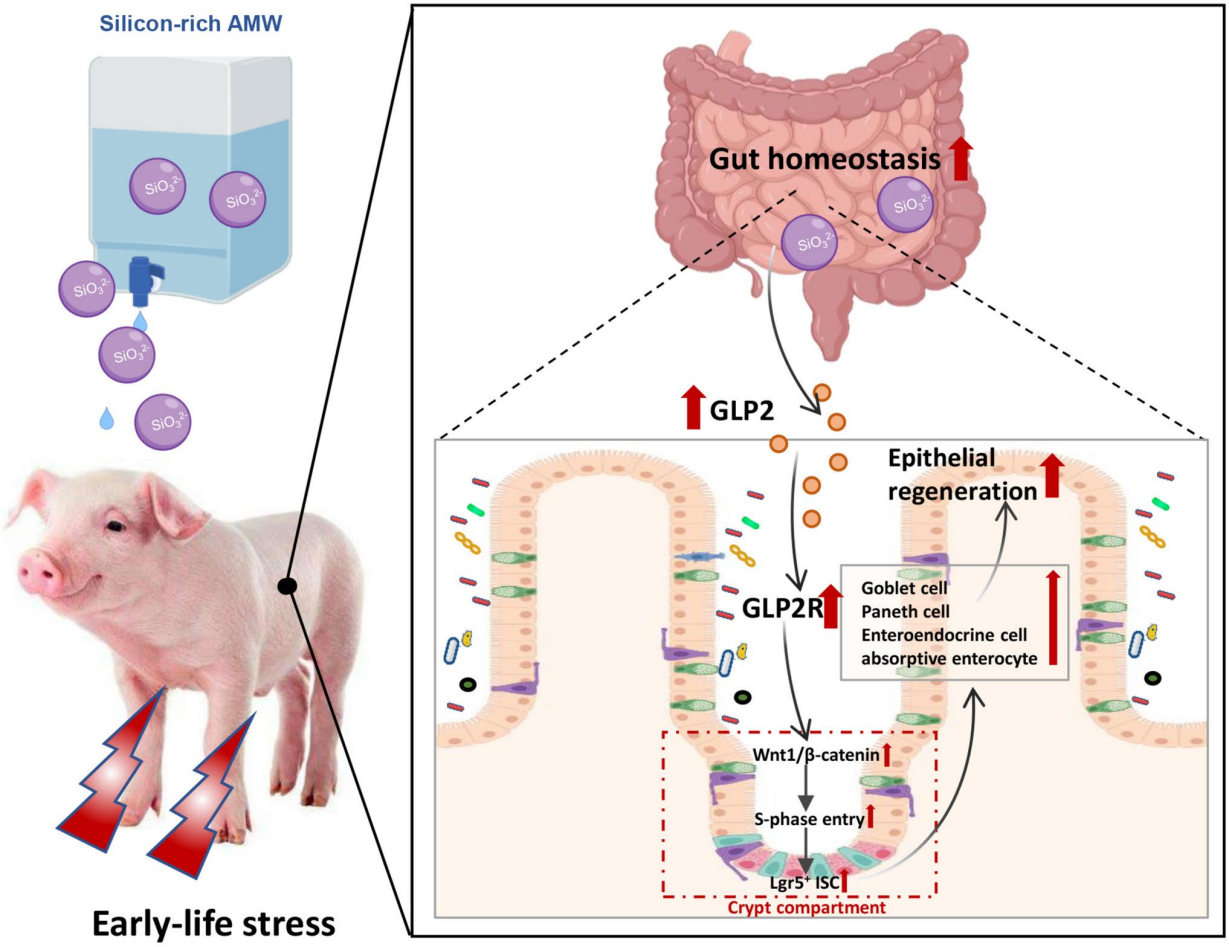
(Graphical abstract). A schematic diagram illustrating the role of GLP2–Wnt1 axis in intestinal epithelium regeneration in piglets under early-life stress. Drinking silicon-rich alkaline mineral water activates GLP2-dependent Wnt1/β-catenin signaling, and drives ISC proliferation and differentiation by stimulating Lgr5^+^ ISC cell cycle passage through the G1–S-phase checkpoint, thereby maintaining intestinal epithelial regeneration

In summary, our findings revealed that drinking silicon-rich AMW maintains intestinal epithelium regeneration through the GLP2–Wnt1 axis in piglets under ELS. Mechanistically, silicon-rich AMW activates GLP2/GLP2R-dependent Wnt1/β-catenin signaling, which drives ISC proliferation and differentiation by stimulating Lgr5^+^ ISC cell cycle passage through the G1–S-phase checkpoint, thereby maintaining intestinal epithelial regeneration (Fig. 7). Our research contributes to the understanding the mechanism of silicon-rich AMW promoting gut epithelial regeneration and provides a new strategy for the treatment of ELS-induced IEI.

### Supplementary Information

Below is the link to the electronic supplementary material.

Supplementary file1 (DOCX 2365 KB)

## Data Availability

All data generated or analyzed during this study are included in this published article.

## Abbreviations

AMW: Alkaline mineral water
APC: Adenomatous polyposis coli
CD: Crypt depth
CDK: Cyclin-dependent kinase
CgA: Chromogranin A
DEFB: Beta-defensin
DEGs: Differentially expressed genes
Edu: 5-Ethynyl-2′-deoxyuridine
ELS: Early-life stress
GBM: Gut–brain–microbiome
GC: Goblet cells
Gcg: Glucagon
GLP: Glucagon-like peptide
GLP2R: GLP2 receptor
HE: Hematoxylin and eosin
HPA: Hypothalamus–pituitary–adrenocortical
IBS: Irritable bowel syndrome
IEC: Intestinal epithelial cells
IEI: Intestinal epithelial injury
IF: Immunofluorescence
Lgr5: Leucine-rich repeat-containing G-protein-coupled receptor 5
LPS: Lipopolysaccharide
MCM: Mini chromosome maintenance
MS: Maternal separation
Muc: Mucin
Nts: Neurotensin
PAS: Periodic acid Schiff
PC: Paneth cells
PCNA: Proliferating cell nuclear antigen
PYY: Peptide YY
qRT-PCR: Quantitative real-Time PCR
RNA-Seq: RNA-sequencing
SEM: Scanning electron microscopy
SI: Small intestine
SiRNA: Small interfering RNA
SMP: Sodium metasilicate pentahydrate
TEM: Transmission electron microscopy
TER: Transepithelial electrical resistance
VH: Villous height
5-HT: 5-Hydroxy-tryptamine
