# Supplementary material for: Role of the GLP2–Wnt1 axis in silicon-rich alkaline mineral water maintaining intestinal epithelium regeneration in piglets under early-life stress
Source: Cell Mol Life Sci. 2024 Mar 12;81(1):126. doi: 10.1007/s00018-024-05162-x (PMC10933158; doi:10.1007/s00018-024-05162-x)
Supplement: Supplementary file 1 — Supplementary file1 (DOCX 2365 KB) [file 18_2024_5162_MOESM1_ESM.docx]

***Supporting Information***

**Role of the GLP2-Wnt1 axis in silicon-rich alkaline mineral water maintaining intestinal epithelium regeneration in piglets under early-life stress**

Jian Chen^a^, Xue-Yan Dai^a^, Bi-Chen Zhao^a^, Xiang-Wen Xu^a^, Jian-Xun Kang^a^, Ya-Ru Xu^a^, Jin-Long Li^a,b,c,*^

^a^College of Veterinary Medicine, Northeast Agricultural University, Harbin 150030, P.R. China; ^b^Key Laboratory of the Provincial Education Department of Heilongjiang for Common Animal Disease Prevention and Treatment, Northeast Agricultural University, Harbin 150030, P.R. China;

^c^Heilongjiang Key Laboratory for Laboratory Animals and Comparative Medicine, Northeast Agricultural University, Harbin 150030, P.R. China

***Corresponding author**

**Jin-Long Li**

Address: College of Veterinary Medicine, Northeast Agricultural University, Harbin, 150030, P. R. China

Tel: +86 45 155190407; fax: +86 451 55190407. E-mail address: Jinlongli@neau.edu.cn (J.L. Li)

**Supplemental Contents**

Materials and methods of RNA-Seq

Supplemental Table 1

Supplemental Table 2

Supplemental Figure 1

Supplemental Figure 2

Supplemental Figure 3

Supplemental Figure 4

**Materials and methods of** **RNA-Seq**

*Sample collection and preparation*

- RNA degradation and contamination was monitored on 1% agarose gels.
- RNA purity was checked using the NanoPhotometer® spectrophotometer (IMPLEN, CA, USA).
- RNA integrity was assessed using the RNA Nano 6000 Assay Kit of the Bioanalyzer 2100 system (Agilent Technologies, CA, USA)

*Library preparation for RNA-Seq sequencing*

- A total amount of 1 µg RNA per sample was used as input material for the RNA sample preparations. Sequencing libraries were generated using NEBNext® UltraTM RNA Library Prep Kit for Illumina® (NEB, USA) following manufacturer’s recommendations and index codes were added to attribute sequences to each sample.
- Briefly, mRNA was purified from total RNA using poly-T oligo-attached magnetic beads. Fragmentation was carried out using divalent cations under elevated temperature in NEBNext First Strand Synthesis Reaction Buffer(5X). First strand cDNA was synthesized using random hexamer primer and M-MuLV Reverse Transcriptase (RNase H-). Second strand cDNA synthesis was subsequently performed using DNA Polymerase I and RNase H. Remaining overhangs were converted into blunt ends via exonuclease/polymerase activities. After adenylation of 3’ ends of DNA fragments, NEBNext Adaptor with hairpin loop structure were ligated to prepare for hybridization. In order to select cDNA fragments of preferentially 250~300 bp in length, the library fragments were purified with AMPure XP system (Beckman Coulter, Beverly, USA). Then 3 µl USER Enzyme (NEB, USA) was used with size-selected, adaptor-ligated cDNA at 37°C for 15 min followed by 5 min at 95℃ before PCR. Then PCR was performed with Phusion High-Fidelity DNA polymerase, Universal PCR primers and Index (X) Primer. At last, PCR products were purified (AMPure XP system) and library quality was assessed on the Agilent Bioanalyzer 2100 system.

*Clustering and sequencing*

- The clustering of the index-coded samples was performed on a cBot Cluster. Generation System using TruSeq PE Cluster Kit v3-cBot-HS (Illumia) according to the manufacturer’s instructions. After cluster generation, the library preparations were sequenced on an Illumina Novaseq platform and 150 bp paired-end reads were generated.

*Data Analysis*

- Quality control: Raw data (raw reads) of fastq format were firstly processed through in-house perl scripts. In this step, clean data (clean reads) were obtained by removing reads containing adapter, reads containing ploy-N and low quality reads from raw data. At the same time, Q20, Q30 and GC content the clean data were calculated. All the downstream analyses were based on the clean data with high quality.
- Reads mapping to the reference genome: Reference genome and gene model annotation files were downloaded from genome website directly. Index of the reference genome was built using Hisat2 v2.0.5 and paired-end clean reads were aligned to the reference genome using Hisat2 v2.0.5.
- Differential expression analysis: Differential expression analysis of two conditions/groups (two biological replicates per condition) was performed using the DESeq2 R package (1.16.1). DESeq2 provide statistical routines for determining differential expression in digital gene expression data using a model based on the negative binomial distribution. The resulting P-values were adjusted using the Benjamini and Hochberg’s approach for controlling the false discovery rate. Genes with an adjusted P-value <0.05 found by DESeq2 were assigned as differentially expressed.
- KEGG enrichment analysis of differentially expressed genes: KEGG is a database resource for understanding high-level functions and utilities of the biological system, such as the cell, the organism and the ecosystem, from molecular-level information, especially large-scale molecular datasets generated by genome sequencing and other high-through put experimental technologies (http://www.genome.jp/kegg/). We used clusterProfiler R package (3.4.4) to test the statistical enrichment of differential expression genes in KEGG pathways.
- GSEA: the enrichment analysis was performed on these expressed data using GSEA software (V4.1.0). In this process, the KEGG gene sets (c2.cp.kegg.v7.0.symbols.gmt) was selected as the functional gene set, other parameters as the default settings. The pathway of gene enrichment with a normal p-value<0.05 and FDR q-value<0.25 has the significance of the statistics.

**Table S1. key resources used in this study**

| **REAGENT or RESOURCE** | **SOURCE** | **IDENTIFIER** |
| --- | --- | --- |
| **Critical Commercial Assays** |  |  |
| Peptide YY | Chenglin | AD10522 |
| 5-hydroxy-tryptamine | Chenglin | AD11646 |
| Neurotensin | Chenglin | AD9829 |
| GLP1 | Chenglin | AD0002 |
| GLP2 | Chenglin | AD0165 |
| Hematoxylin-Eosin/HE Staining Kit | bioss | C02-04004 |
| Periodic Acid Schiff (PAS) Stain Kit | Solarbio | G1281 |
| Cell Cycle Analysis Kit | Beyotime | C1052 |
| BeyoClick™ EdU Cell Proliferation Kit | Beyotime | C0071S |
| Mitochondrial membrane potential assay kit with JC-1 | Beyotime | C2006 |
| Cell Counting Kit-8 | Dojindo | CK04 |
| **Main chemicals** |  |  |
| RNAout | TIANGEN | DP431 |
| Normal Goat Serum | bioss | C-0005 |
| Paraformaldehyde, 4% | bioss | C01-06002 |
| DMEM/F12 | Gibco | 11330-032 |
| FBS | HyClone | SV30208.02 |
| Opti-MEM | Invitrogen | 11058021 |
| DAPI | Beyotime | C1005 |
| LPS | Sigma | L3012 |
| GLP2 | MedChemExpress | 223460-79-5 |
| GLP2^3-33^ | MedChemExpress | 275801-62-2 |
| Lipofectamine 2000 | Thermo Fisher | 12566014 |
| Transwell | Corning | C3460 |
| **Antibodies** |  |  |
| Gcg | Santa Cruz | sc-514592 |
| TGR5 | Abclonal | A20778 |
| GLP2R | Abclonal | A6602 |
| Wnt1 | Affinity | AF5315 |
| Lrp6 | Affinity | DF2995 |
| β-catenin | Abclonal | A19657 |
| Axin | bioss | bs-21731R |
| GSK-3β | bioss | bs-0028R |
| APC | Affinity | AF9039 |
| PCNA | Abclonal | A9909 |
| Ki67 | Affinity | AF0198 |
| Lgr5 | bioss | bs-20746R |
| Cyclin D | Affinity | AF0931 |
| Cyclin E | Wanlei | WL01072 |
| CDK2 | Abclonal | A0294 |
| CDK6 | proteintech | 66278-1-Ig |
| MCM2 | proteintech | 66204-1-Ig |
| Lys | Abclonal | A13511 |
| CgA | proteintech | 10529-1-AP |
| Muc2 | Abclonal | A14659 |
| β-actin | Abclonal | AC026 |
| HRP-conjugated secondary antibody | bioss | bs-40295G-HRP |
| Goat Anti-Rabbit IgG H&L (Alexa Fluor® 488) | abcam | ab150077 |
| Goat Anti-Rabbit IgG H&L (Alexa Fluor® 594) | abcam | ab150080 |
| Goat Anti-Mouse IgG H&L (Alexa Fluor® 488) | abcam | ab150113 |

**Table S2.** **Primers used for qRT-PCR analysis in this study**

| Gene name | Accession number | Primer and probe sequences (5′ to 3′) |
| --- | --- | --- |
| *GAPDH1* | NM_001206359.1 | F: TCGGAGTGAACGGATTTGGC  R: TGACAAGCTTCCCGTTCTCC |
| *GAPDH2* | NM_001206359.1 | F: CGGAGTGAACGGATTTGGC  R: CACCCCATTTGATGTTGGCG |
| *Gcg* | NM_214324.1 | F: ATGACTGAAGACAAGCGCCA |
|  |  | R: AGTGACATACTTCCTAGAGACTGT |
| *TGR5* | XM_013984487.2 | F: CCATGCACCCCTGTTGCT |
|  |  | R: GGTGCTGTTGGGTGTCATCTT |
| *GLP1R* | NM_001256594.1 | F: TTTTGCCATCGGGGTGAACT |
|  |  | R: TTGGTGTCCGTCTTGCACAT |
| *GLP2R* | NM_001246266.1 | F: CCCTGCTGTTTCTGGTTTCC |
|  |  | R: GGCAGGGAACAGAAACGTTT |
| *CDK4* | NM_001123097.1 | F: CTTTGCTGAGATGGTGACCCG |
|  |  | R: GCTGAAATCCGCTTGTTGGG |
| *S100A6* | NM_001044557.1 | F: TGATCTGGACCGGAACAAGG |
|  |  | R: CCCGGAGGACATCGTTGTAG |
| *MCM2* | XM_003483239.4 | F: CCGCCTTGACGAAAATGGAC |
|  |  | R: GGACTCAGATGATTCCGCCA |
| *MCM3* | XM_001924813.6 | F: TGAGCGAGTCCAGGTTGAAG |
|  |  | R: AAGGCAAATACTGGGGGAGC |
| *MCM4* | XM_021089418.1 | F: TGCACGTCACAGGCATCTAC |
|  |  | R: GGCTGAAGCAAGTCTCTCGT |
| *MCM5* | XM_021081469.1 | F: TCCAAACCGCAGCGTCTATT |
|  |  | R: CCGCTTGAGTTCATCCCTGT |
| *MCM6* | XM_021075844.1 | F: ACCAACCCAAGGTTTGGAGG |
|  |  | R: TAGCTGTACTCGGGTCACCA |
| *MCM7* | XM_021086193.1 | F: GGACCTCCTCTCTGATTGGC |
|  |  | R: CGATGAGCCAATCGAACCAAC |
| *Sucrase-Isomaltase* | XM_021069748.1 | F: CACCTGCTGTTGAAGAAATTAGT |
|  |  | R: CATTCCATGGCTTCCAGCAG |
| *Lys1* | NM_214392.2 | CCCAGCACCCAGTTAGAACA |
|  |  | GGCACAGCTCACTAGTCCTC |
| *DEFB1* | NM_213838.1 | F: CCACCAGCATGAGACTCCAC |
|  |  | R: TTGCAGCATTTGACTTGGGG |
| *DEFB2* | NM_010030.2 | F: CCAGCTGGCTGCAGGTATTA |
|  |  | R: ACTTGGCCTTGCCACTGTAA |
| *Muc1* | XM_021089730.1 | F: GTGCCGACGAAAGAACTG |
|  |  | R: TGCCAGGTTCGAGTAAGAG |
| *Muc2* | XM_021082584.1 | F: CTGTGTGGGGCCTGACAA |
|  |  | R: AGTGCTTGCAGTCGAACTCA |


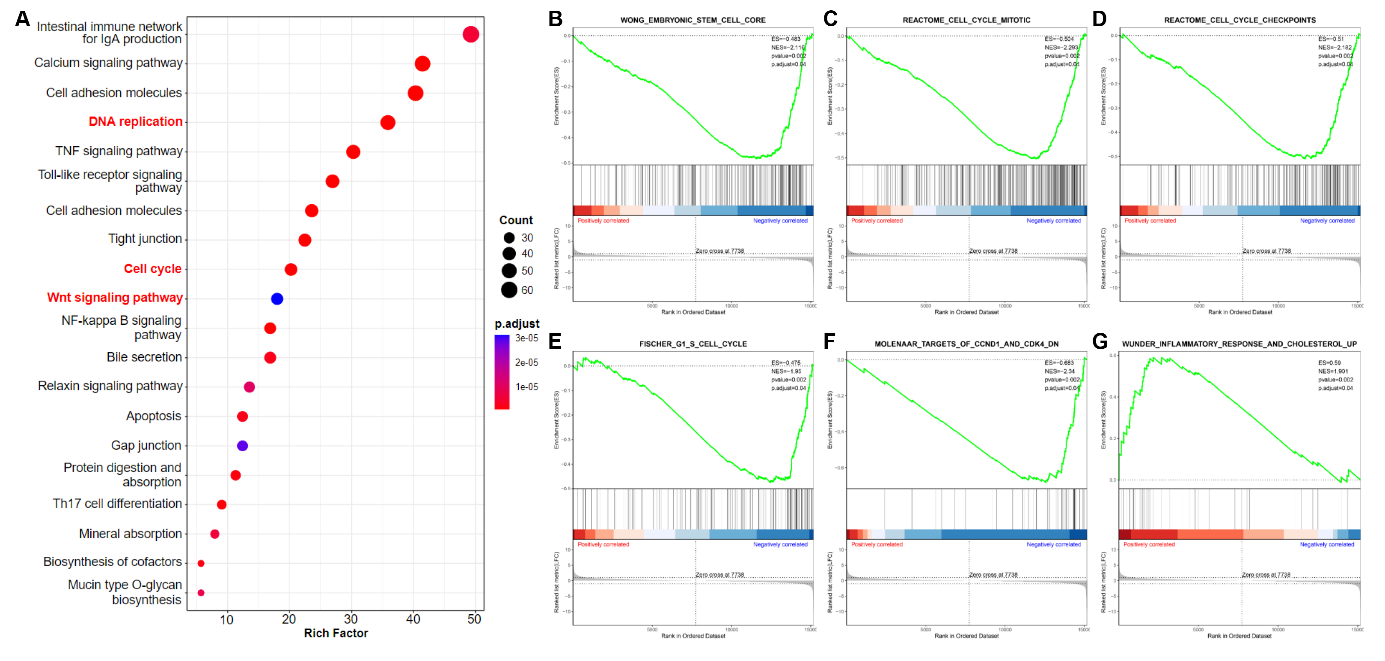
 **Fig. S1 Analysis of DEGs in Con vs SMP groups.** (A) KEGG analysis based on RNA-seq in the jejunum of MS piglets after drinking silicon-rich alkaline mineral water. (B-G) Gene set enrichment analysis (GSEA). NES: normalized enrichment score; p, nominal p-value; q, false discovery rate q-value. FDR: false discovery rate. It is generally considered that the gene set under the pathway of |NES|> 1, NOM p-value < 0.05, and FDR q-value < 0.25 is meaningful. The larger the absolute value of NES, the smaller the FDR value, and the higher credibility of the analysis results. The NOM p-value represents the statistical significance of the enrichment score (ES) value of a functional gene set. The smaller the p-value is, the better the gene enrichment is.


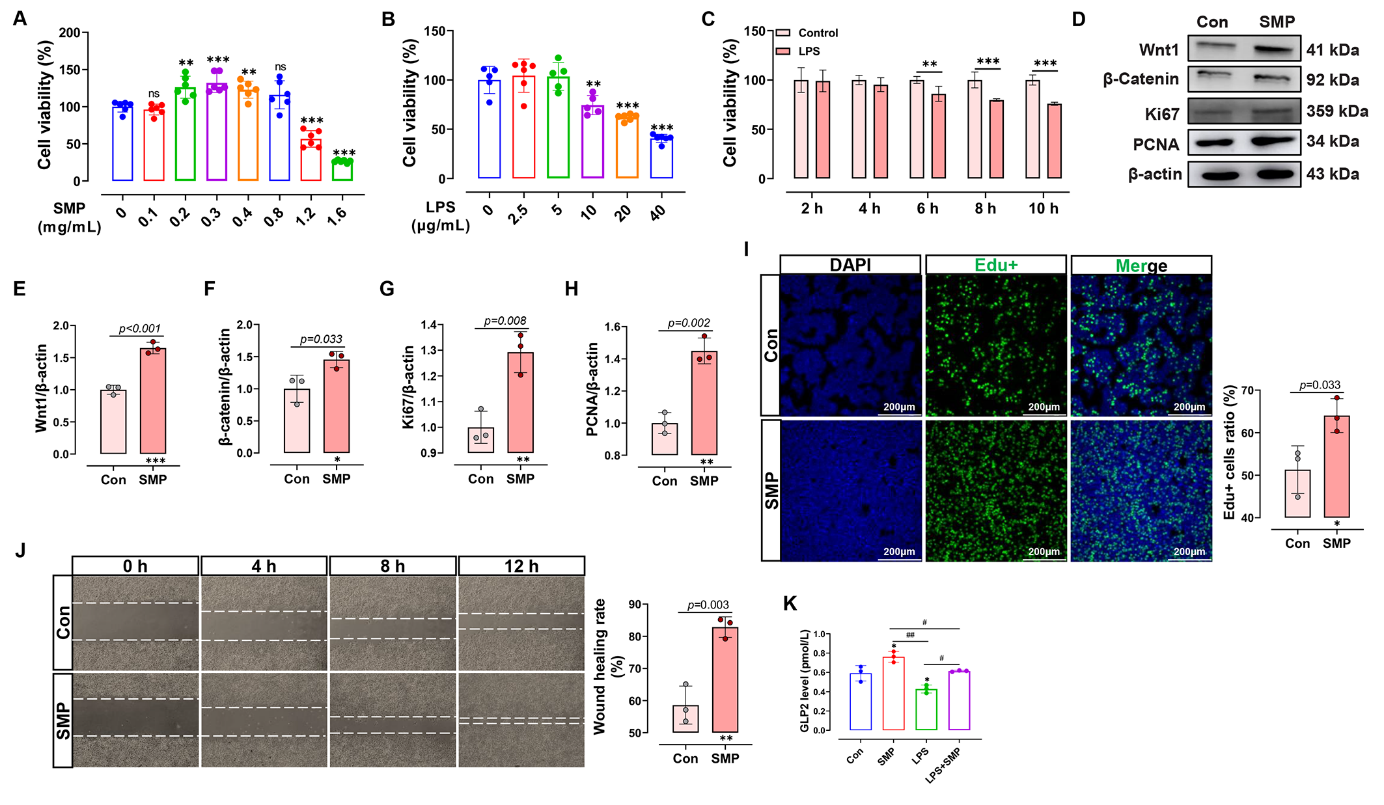


**Fig. S2 SMP promoted IPEC-J2 cell regeneration by activating Wnt1/β-catenin signaling under physiological condition.** (A-C) SMP treatment concentration and LPS injury model establishment in IPEC-J2 cells through CCK8 cell viability assay. (A) Cell viability in various concentrations of SMP (0, 0.1, 0.2, 0.3, 0.4, 0.8, 1.2 and 1.6 mg/mL) treatment. (B) Cell viability in various concentrations of LPS (0, 2.5, 5, 10, 20 and 40 μg/mL) exposure. (C) Cell viability at 2, 4, 6, 8 and 10 h after 10 μg/mL LPS exposure. (D) Western blot analysis of Wnt1, β-catenin, Ki67 and PCNA protein expression. (E-H) The relative protein levels of Wnt1, β-catenin, Ki67 and PCNA in the IPEC-J2 cells under physiological condition. (I) Immunofluorescence images and statistical analysis of Edu+ (green) staining and DAPI (blue) in the IPEC-J2 cells under physiological condition. (J) Wound healing rate in the IPEC-J2 cells under physiological condition. (K) GLP2 expression level in the IPEC-J2 cells after SMP and/or LPS treatment. Data are presented as the mean ± SD. Statistical analysis was calculated using Student’s t-tests to compare differences between the two groups or one-way ANOVA for multiple group comparison followed by Tukey’s post hoc pairwise comparison. ^ns^*P*>0.05, **P*<0.05, ***P*<0.01, and ****P*<0.001. ^#^*P*<0.05 and ^##^*P*<0.01.


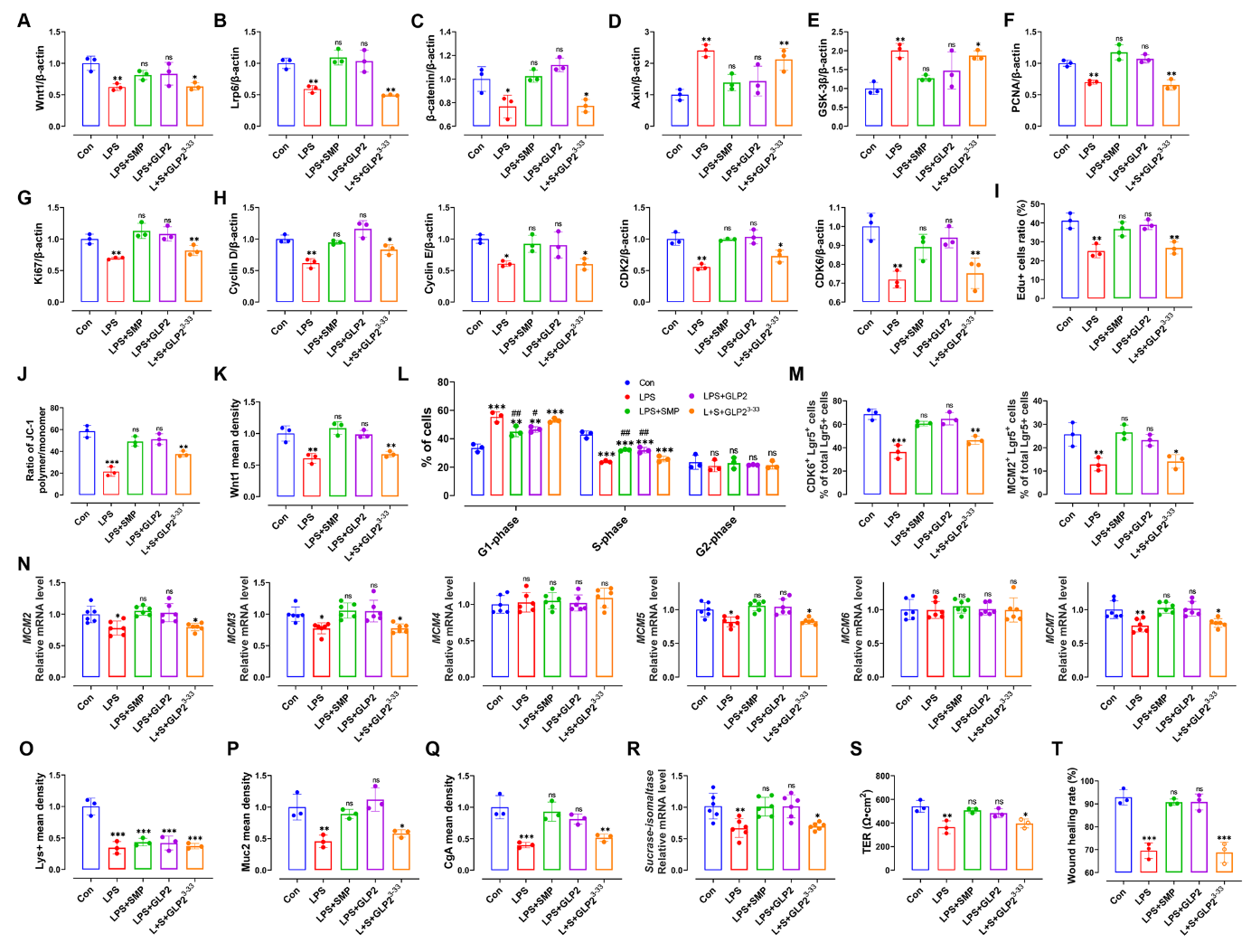
 **Fig. S3 Inhibition of GLP2/GLP2R signaling by GLP23-33 disrupted the regeneration effect of SMP in LPS-Challenged IPEC-J2 Cells.** (A-G) The relative protein levels of Wnt1, Lrp6, β-catenin, Axin, GSK-3β, APC, PCNA and Ki67 in LPS-challenged IPEC-J2 cell. (H) The relative protein levels of Cyclin D, Cyclin E, CDK2 and CDK6 in LPS-challenged IPEC-J2 cell. (I) Statistical analysis of Edu^+^ cells ratio. (J) Statistical analysis of JC-1 polymer/monomer ratio. (K) Statistical analysis of Wnt1 mean density. (L) Statistical analysis of G1-phase, S-phase and G2-phase cell cycle in LPS-challenged IPEC-J2 cell. (M) Statistical analysis of CDK6^+^Lgr5^+^ cells % and MCM2^+^Lgr5^+^ cells % of total Lgr5+ cell. (N) The relative mRNA levels of MCM2, MCM3, MCM4, MCM5, MCM6 and MCM7 in LPS-challenged IPEC-J2 cell. (O-Q) Statistical analysis of Lys, Muc2 and CgA mean density. (R) Statistical analysis of sucrase-isomaltase mRNA level. (S) Statistical analysis of TER in LPS-challenged IPEC-J2 cell. (T) Statistical analysis of wound healing rate. Data are presented as the mean ± SD. Statistical analysis was calculated using one-way ANOVA for multiple group comparison followed by Tukey’s post hoc pairwise comparison. ^ns^*P*>0.05, **P*<0.05, ***P*<0.01, and ****P*<0.001 vs. the Con group.


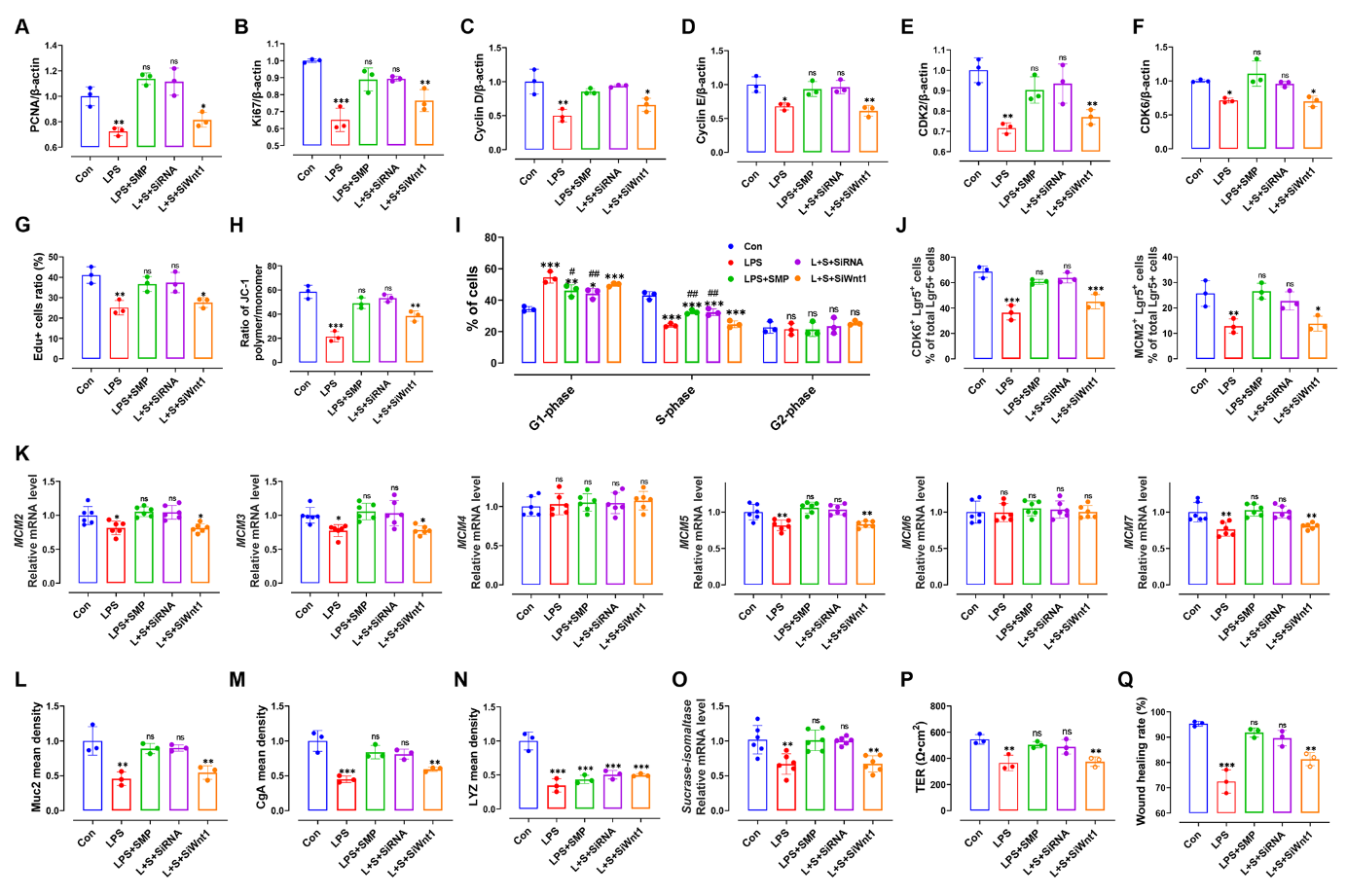


**Fig. S4 Silencing Wnt1 by SiRNA blunted SMP-stimulated IPEC-J2 cell regeneration.** (A-F) The relative protein levels of PCNA, Ki67, Cyclin D, Cyclin E, CDK2 and CDK6 in LPS-challenged IPEC-J2 cell after silencing Wnt1. (G) Statistical analysis of Edu^+^ cells ratio. (H) Statistical analysis of JC-1 polymer/monomer ratio. (I) Statistical analysis of G1-phase, S-phase and G2-phase cell cycle in LPS-challenged IPEC-J2 cell after silencing Wnt1. (J) Statistical analysis of CDK6^+^Lgr5^+^ cells % and MCM2^+^Lgr5^+^ cells % of total Lgr5+ cell. (K) The relative mRNA levels of MCM2, MCM3, MCM4, MCM5, MCM6 and MCM7 in LPS-challenged IPEC-J2 cell. (L-N) Statistical analysis of Muc2, CgA and Lys mean density. (O) Statistical analysis of sucrase-isomaltase mRNA level. (S) Statistical analysis of TER in LPS-challenged IPEC-J2 cell after silencing Wnt1. (T) Statistical analysis of wound healing rate. Data are presented as the mean ± SD. Statistical analysis was calculated using one-way ANOVA for multiple group comparison followed by Tukey’s post hoc pairwise comparison. ^ns^P>0.05, *P<0.05, **P<0.01, and ***P<0.001 vs. the Con group.
